# Splenic abscess with spontaneous gastrosplenic fistula: case report and review of the literature

**DOI:** 10.1093/jscr/rjad647

**Published:** 2023-12-05

**Authors:** Andrea Campisi, Lorenzo Rocca, Gaia Altieri, Valeria Fico, Gilda Pepe, Claudio Lodoli

**Affiliations:** Fondazione Policlinico Universitario Agostino Gemelli, Catholic University, Largo Francesco Vito 1, 00168 Rome, Italy; Fondazione Policlinico Universitario Agostino Gemelli, Catholic University, Largo Francesco Vito 1, 00168 Rome, Italy; Fondazione Policlinico Universitario Agostino Gemelli, Catholic University, Largo Francesco Vito 1, 00168 Rome, Italy; Fondazione Policlinico Universitario Agostino Gemelli, Catholic University, Largo Francesco Vito 1, 00168 Rome, Italy; Fondazione Policlinico Universitario Agostino Gemelli, Catholic University, Largo Francesco Vito 1, 00168 Rome, Italy; Fondazione Policlinico Universitario Agostino Gemelli, Catholic University, Largo Francesco Vito 1, 00168 Rome, Italy

**Keywords:** gastrosplenic fistula, splenic abscess, surgical resection, melena, case report

## Abstract

Gastrosplenic fistula (GSF) is an unusual event that might occur in patients with various gastric or splenic diseases. While GSF related to gastric and splenic malignancies is well-documented in the literature, cases of GSF due to a splenic abscess are extremely rare. We experienced the case of a 49-year-old man with a medical history of tricuspid cardiac valve replacement for infective endocarditis who presented with a sudden onset of anemia and melena. With the assistance of imaging and endoscopy, a primary splenic abscess complicated by spontaneous GSF was diagnosed. A prompt splenectomy with partial gastrectomy was performed. GSF is a serious occurrence associated with a high risk of morbidity and mortality. The early recognition of GSF related to a splenic abscess is crucial to prevent major complications. Surgical resection with splenectomy and partial gastrectomy is frequently preferred for the treatment of large abscesses with GSF.

## Introduction

Gastrosplenic fistula (GSF) is a rare occurrence that primarily arises as a complication of gastric or splenic malignant tumors [1]. Although there are few reported cases of GSF associated with peptic ulcers and Crohn’s disease, instances of GSF originating from benign spleen conditions are exceptionally uncommon [1]. GSF is linked to significant clinical implications and may necessitate prompt embolization or surgical intervention in case of severe bleeding [2]. To prevent major complications, the standard treatment approach for patients with GSF caused by a splenic abscess involves splenectomy with partial gastric resection. This article aims to describe a unique clinical case of GSF resulting from a splenic abscess and provide a literature review on GSF as a complication of benign splenic pathologies.

## Case report

The patient is a 49-year-old Caucasian male with medical history of HCV-HBV infections, substance use disorder, and tricuspid cardiac valve replacement using a bioprosthetic valve due to complications from infective endocarditis. Subsequently, a left-brain hemisphere stroke and splenic embolizations occurred.

**Figure 1:**
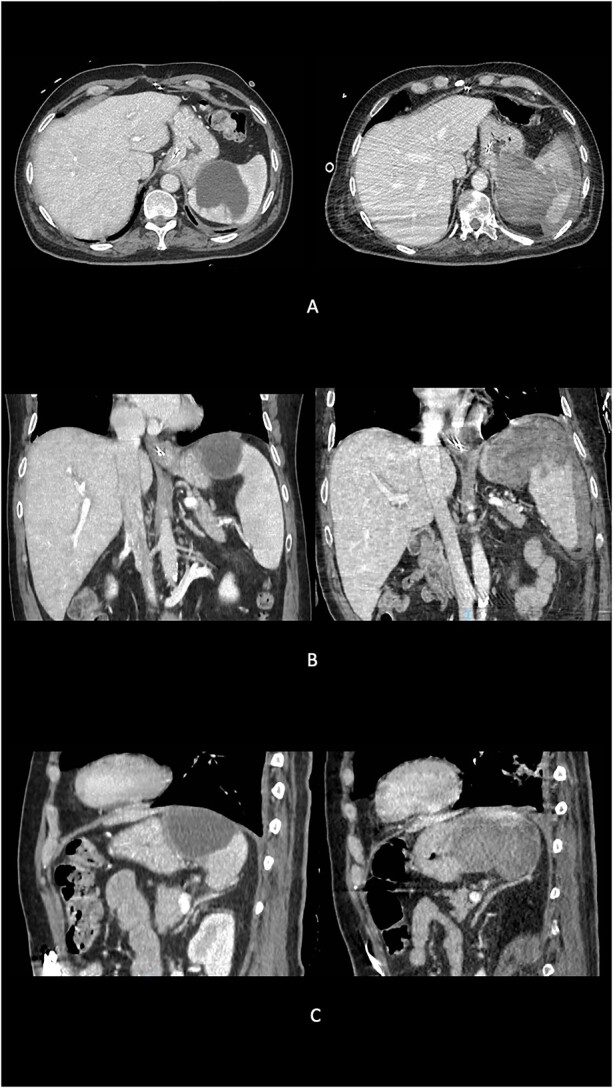
Preoperative evolution of splenic abscess into GSF at imaging. (A) Axial scans. (B) Coronal scans. (C) Sagittal scans.

A transesophageal echocardiogram revealed hyperechoic images in both mitral valve leaflets with medium-grade valve insufficiency and tricuspid dysfunction. Peripheral venous blood cultures yielded negative results, and empiric antibiotic therapy was initiated. Infective mitral valve endocarditis was staged using an abdominal computed tomography (CT) scan with contrast medium, which identified an 8 × 7 cm neoformation at the upper pole of the spleen, consistent with an abscess lesion (Fig. 1).

Two weeks later, an endoscopic ultrasound was performed, revealing an abscess collection measuring 7 × 6 cm in the upper pole of the spleen. The portion of the collection adjacent to the splenic parenchyma did not appear to be encapsulated, and drainage of the abscess using a metal prosthesis would likely have caused pressure on healthy splenic tissue.

As a result, the patient underwent six consecutive abdominal ultrasounds, which demonstrated a gradual reduction in the size of the splenic collection (minimum diameter: 4 cm).

Three months after the diagnosis of splenic abscess, the patient experienced a sudden onset of anemia and melena. An abdominal CT scan revealed a large blood collection (maximum axial diameter of 10 cm, cranio-caudal extension of 15 cm) replacing the upper pole of the splenic parenchyma (Fig. 1). The splenic capsular profile was disrupted, and the blood collection extended medially, affecting the gastric fundus wall and entering its lumen.

**Figure 2:**
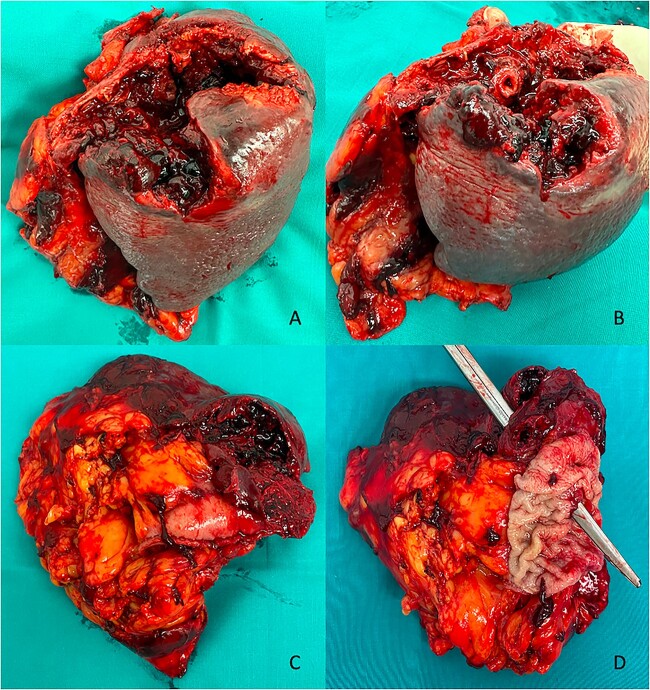
Postoperative macroscopic view. (A) Anterior view of the splenic blood–abscess collection. (B) Anterior view of the GSF. (C) Posterior view of the splenic blood–abscess collection with the gastric stump. (D) Posterior view of the GSF.

An exploratory laparotomy was performed, revealing numerous clots around the spleen and a large splenic abscess with inflammatory adhesions to the gastric fundus and left diaphragm. En block resection of the spleen along with a portion of the gastric fundus was carried out (Fig. 2).

Histopathological examination (HPE) showed a splenic blood–abscess collection with GSF and adhesive perisplenitis involving adjacent tissues, but no evidence of malignancy.

The postoperative period was uneventful.

## Review of the literature

To our knowledge, there have been six reported cases of GSF resulting from benign splenic diseases [3–8]. Among these cases, four patients presented with splenic abscess, as shown in Table 1, one patient had splenic tuberculosis, and one patient developed posttraumatic GSF.

**Table 1:** A summary of the articles describing the surgical management of GSF.

**Author**	**Year**	**Age/gender**	**Presenting symptom**	**Diagnosis**	**Surgical treatment**	**Post-surgery HPE**
Kryshtalskyj N. *et al*.	1991	46/woman	Left flank pain with left shoulder irradiation and fever	CT + gastroscopy	Splenectomy and partial gastrectomy	GSF, one infarct, two splenic abscesses
Ballas K *et al*.	2005	70/man	Fatigue, anorexia, weight loss, and epigastric postprandial fullness	CT + gastroscopy	Splenectomy and partial gastrectomy	GSF, extensive infiltration of the stomach wall with neutrophils, eosinophils, lymphocytes, and plasma cells
Leeds IL *et al*.	2015	79/woman	Left upper quadrant pain, general malaise, dyspnea, and occasional dizziness	CT + gastroscopy	Splenectomy, partial gastrectomy, and distal pancreatectomy	GSF, diffuse B-cell lymphoma
Alsinan A *et al*.	2019	15/man	Left-sided abdominal pain, anemia	CT + gastroscopy	Splenectomy and partial gastrectomy	GSF, splenic abscess
Campisi A. *et al*.	2023	49/man	Anemia, melena	CT + gastroscopy	Splenectomy and partial gastrectomy	GSF, splenic abscess, and adhesive perisplenitis to perilous tissue

Kryshtalskyj *et al*. [3] described the first reported case of GSF caused by a splenic abscess, which was treated with splenectomy and partial gastrectomy. No predisposing factors for abscess were identified. The postoperative HPE revealed GSF along with one splenic infarct and two splenic abscesses.

Ballas *et al*. [4] reported a rare case of GSF in a severely malnourished patient with a splenic abscess. Serological tests showed no evidence of infection, and there were no signs of endocarditis or immunodeficiency. CT scan revealed communication between the stomach, an intra-abdominal abscess, and the spleen. The HPE did not reveal any significant splenic pathological abnormalities, suggesting that fistula originated from the stomach.

Leeds *et al*. [5] published a case report of a 79-year-old frail woman with GSF and splenic abscess who underwent excisional surgery. Intraoperative findings showed a splenic mass with GSF, abscess, and inflammatory adhesions to the stomach, distal pancreas, and left diaphragm. The patient underwent splenectomy, partial gastrectomy, and distal pancreatectomy. The postoperative HPE confirmed the presence of diffuse B-cell lymphoma.

Alsinan *et al*. [6] described the case of a 15-year-old patient with sickle cell trait who developed a splenic abscess following a sequestration crisis. The abscess progressed to GSF, causing left-sided abdominal pain and acute anemia. The patient underwent open splenectomy and partial gastrectomy.

## Discussion

GSF is a serious complication that can arise from various gastric and splenic diseases, often requiring prompt embolization or surgical intervention in cases of significant bleeding [2]. Most commonly, GSF occurs as a result of tumor progression in splenic B-cell lymphoma or chemotherapy for advanced-stage disease [9, 10]. However, the development of GSF in patients with benign splenic diseases is a very rare occurrence, typically happening spontaneously [1, 2].

GSF often does not present with severe hemorrhage, and short-term outcomes are generally favorable, with a survival rate of approximately 82% [2]. However, massive bleeding is a severe complication of GSF associated with significant mortality [2].

The diagnosis of GSF is typically suspected based on CT imaging, which reveals air in the spleen, and is confirmed through upper gastrointestinal endoscopy, allowing direct visualization of the fistulous communication [2].

The treatment approach should consider the underlying cause of GSF. However, it can be challenging to determine whether GSF is a result of silent gastric perforation with secondary splenic abscess or a primary splenic abscess leading to the formation of a communication with the stomach.

Conservative treatment with percutaneous drainage and antibiotic therapy is often inadequate for GSF patients with large splenic abscesses. In such cases, the most common treatment is splenectomy with partial gastrectomy, aiming to prevent major complications, including massive bleeding [1].

## Conclusions

GSF is an uncommon complication associated with splenic abscesses. Early recognition of GSF related to splenic abscess is essential to prevent significant complications, such as severe bleeding. Although conservative approaches are possible, surgical resection involving splenectomy and partial gastrectomy is often the preferred treatment for large abscesses with GSF. In such cases, the presence of active bleeding further reinforces the necessity of surgical intervention.

## Data Availability

Research data supporting this publication are available from the corresponding author on reasonable request.
